# Mathematical design of prokaryotic clone-based microarrays

**DOI:** 10.1186/1471-2105-6-238

**Published:** 2005-09-28

**Authors:** Bart Pieterse, Elisabeth J Quirijns, Frank HJ Schuren, Mariët J van der Werf

**Affiliations:** 1 Wageningen Centre for Food Sciences. Diedenweg 20, 6700 AN Wageningen, The Netherlands; 2 TNO Quality of Life. Utrechtseweg 48, 3700 AJ Zeist, The Netherlands; 3 BioDetection Systems, Kruislaan 406, 1098 SM, Amsterdam, The Netherlands; 4 Wageningen University and Research Centre, Systems and Control Group, Department of Agrotechnology and Food Sciences, Bornsesteeg 59, 6708 PD Wageningen, The Netherlands; 5 HAS Den Bosch, Onderwijsboulevard 221, 5200 MA, Den Bosch, The Netherlands

## Abstract

**Background:**

Clone-based microarrays, on which each spot represents a random genomic fragment, are a good alternative to open reading frame-based microarrays, especially for microorganisms for which the complete genome sequence is not available. Since the generation of a genomic DNA library is a random process, it is beforehand uncertain which genes are represented. Nevertheless, the genome coverage of such an array, which depends on different variables like the insert size and the number of clones in the library, can be predicted by mathematical approaches. When applying the classical formulas that determine the probability that a certain sequence is represented in a DNA library at the nucleotide level, massive amounts of clones would be necessary to obtain a proper coverage of the genome.

**Results:**

This paper describes the development of two complementary equations for determining the genome coverage at the gene level. The first equation predicts the fraction of genes that is represented on the array in a detectable way and cover at least a set part (the minimal insert coverage) of the genomic fragment by which these genes are represented. The higher this minimal insert coverage, the larger the chance that changes in expression of a specific gene can be detected and attributed to that gene. The second equation predicts the fraction of genes that is represented in spots on the array that only represent genes from a single transcription unit, which information can be interpreted in a quantitative way.

**Conclusion:**

Validation of these equations shows that they form reliable tools supporting optimal design of prokaryotic clone-based microarrays.

## Background

In the past decade, whole transcriptome comparison by microarray hybridizations has proven to be an effective tool for studying genome wide gene responses. The general approaches for the development of microarrays are based on the completely annotated genome sequence of an organism. Usually each spot on the array represents one open reading frame (ORF). Whereas this approach has clear advantages for strains for which the complete annotated genome sequence is available, it is not applicable to strains for which this is not the case.

A method that allows for the rapid construction of microarrays for which the completely annotated genome sequence is not required is by the construction of a clone-based array. In this approach, a chromosomal DNA library is constructed from the strain of interest. From this library the genomic fragments, the inserts, are amplified from the clones by PCR with generic primers and spotted on the array-slide [1,2].

The major differences between ORF-based and clone-based arrays with respect to the data interpretation are that in case of clone-based arrays the differential signals can only be linked to a specific gene after the DNA fragment from the spot of interest on the array has been sequenced, and that it is beforehand uncertain whether a gene is represented on the array. Moreover, whereas ORF-based microarrays exclusively generate gene specific data, a differential signal within a spot on a clone-based array can originate from multiple genes on the insert that are not necessarily linked at the transcriptional level.

The extent of these limitations can be quantified by estimating the genome coverage by the spots present on the array. The standard formulas for estimating the genome coverage of a DNA library, the Clark-Carbon equation [3] and the Lander-Waterman equation [4], determine this coverage at the nucleotide level. In other words, they consider the genome as a set of nucleotides, which is useful when the library is to be used for genome sequencing. However, these formulas will overestimate the required number of clones for hybridization purposes. The reason for this is that for hybridization purposes small overlapping fragments that allow for specific binding of the labeled cDNA suffice. Akopyants *et al*. [5] developed an equation for the estimation of the fraction of genes that are at least partially represented. This formula is directly derived from classical probability calculations and contains the organism specific variables genome size and average gene size. Due to the fact that Akopyants *et al*. determine the genome coverage at the gene level, and consider a gene represented if a fragment is present that is large enough to hybridize to and large enough to identify the gene, the required number of clones to obtain a certain coverage is reduced.

A general drawback of these three formulas is that they give no insight into the fraction of genes for which specific data can be generated in a transcriptomics experiment. The data from a spot are considered specific if the expression ratios from the quantified signal from that spot can directly be related to the gene(s) represented by the spot. This is not the case if DNA from multiple (neighboring) transcription units is present in one spot, since it would be uncertain which gene is responsible for which part of the total signal from that spot.

In this paper, two formulas were developed that enable for mathematical predictions of genome coverage by a prokaryotic clone based-array at the gene level as a function of genome size, number of clones, insert size, and either the minimal part of the insert that is covered by the gene or the minimal overlap of the gene and the insert: the minimal insert coverage (MIC) equation, and the gene specific information (GSI) equation.

In order to develop equations that are applicable to a broad range of microorganisms, model datasets were generated for 15 prokaryotes originating from several genera (Table 1) that functioned as templates on which the MIC- and GSI-equations were fitted. The resulting formulas were validated on 10 other prokaryotic species.

**Table 1:** Overview of prokaryotes from several genera with their genes/transcription unit-ratio. Microorganisms that were used for model development (M) or validation (V) of the MIC- and the GSI-equation are depicted in the list.

** Genus **	** Organism **	**genes/TU (R)**	**Model (M) or validation (V) strain**
**Proteobacteria Gammaproteobacteria Enterobacteriales**	*Escherichia coli *K-12 MG1655	1.6	
	*Escherichia coli *O157:H7 EDL933	1.6	
	*Escherichia coli *CFT073	1.6	M
	*Salmonella typhi *CT19	1.4	
	*Salmonella typhimurium *LT2	1.6	
	*Yersinia pestis *CO92	1.4	
	*Shigella flexneri *2a str. 2457T	1.5	
	*Buchnera aphidicola *Sg	1.5	V
	*Wigglesworthia glossinidia*	1.5	
**ProteobacteriaGammaproteobacteria Pasteurellales**	*Haemophilus influenzae Rd*	1.7	
	*Pasteurella multocida *PM70	1.7	V
**Proteobacteria Gammaproteobacteria Xanthomonadales**	*Xylella fastidiosa *9a5c	1.5	
	*Xanthomonas campestris *ATCC33913	1.5	V
**Proteobacteria Gammaproteobacteria Vibrionales**	*Vibrio cholerae *El Tor N16961	1.8	M
	*Vibrio parahaemolyticus *RIMD2210633	1.5	
	*Vibrio vulnificus *CMCP6	1.5	
**Proteobacteria Gammaproteobacteria Pseudomonadales**	*Pseudomonas aeruginosa *PA01	1.6	M
	*Pseudomonas putida *KT2440	1.6	
**Proteobacteria Gammaproteobacteria Legionellales**	*Coxiella burnetii *RSA 493	1.6	
**Proteobacteria Betaproteobacteria**	*Neisseria meningitidis *Z2491	1.6	M
	*Ralstonia solanacearum *GMI1000	1.6	
**Proteobacteria Epsilonproteobacteria**	*Helicobacter pylori *26695	2.3	M
	*Campylobacter jejuni *NCTC11168	2.7	M
**Proteobacteria Alphaproteobacteria**	*Rickettsia prowazekii *Nadrid E	1.4	V
	*Sinorhizobium meliloti *1021	1.5	
	*Agrobacterium tumefaciens *C58	1.5	
	*Brucella suis *1330	1.5	
	*Caulobacter crescentus*	1.5	
**Firmicutes Bacillales**	*Bacillus subtilis *168	1.6	M
	*Oceanobacillus iheyensis *HTE831	1.6	
	*Stapylococcus aureus *MW2	1.6	
	*Listeria monocytogenes *EGD-e	1.8	M
	*Listeria innocua *Clip11262	1.8	
**Firmicutes Clostridia**	*Clostridium acetobutylicum *ATCC824	1.6	
	*Clostridium tetani *E88	1.6	
	*Thermoanaerobacter tengcongensis *MB4T	2.0	
**Firmicutes Lactobacillales**	*Lactococcus lactis *IL1403	1.5	M
	*Streptococcus agalactiae *2603	1.8	
	*Streptococcus pneumoniae *R6	1.8	
	*Lactobacillus plantarum *WCFS1	1.6	M
	*Enterococcus faecalis *V583	1.8	
**Firmicutes Mollicutes**	*Mycoplasma pneumoniae *M129	2.1	M
	*Mycoplasma genitalium *G37	3.1	V
	*Mycoplasma penetrans *HF-2	1.6	
	*Ureaplasma urealyticum *(serovar 3)	2.1	
**Actinobacteria**	*Mycobacterium tuberculosis *H37Rv	1.7	M
	*Corynebacterium glutamicum *ATCC 13032	1.5	
	*Streptomyces coelicolor *A3(2)	1.4	
	*Tropheryma whipplei *Twist	1.9	
	*Bifidobacterium longum *NCC2705	1.3	V
**Fusobacteria**	*Fusobacterium nucleatum *ATCC25586	2.0	V
**Chlamydia**	*Chlamydia trachomatis *(serovar D)	1.6	
	*Chlamydophila pneumoniae *AR39	1.6	
**Spirochete**	*Borrelia burgdorferi *B31	1.8	
	*Treponema pallidum *Nichols	1.9	
	*Leptospira interrogans *56601	1.5	
**Bacteroid**	*Bacteroides thetaiotaomicron *VPI-5482	1.8	M
**Cyanobacteria**	*Thermosynechococcus elongatus *BP-1	1.6	
	Nostoc sp. PCC 7120	1.2	
**Green sulfur bacteria**	*Chlorobium tepidum *TLS	1.6	
**Deinococcus**	*Deinococcus radiodurans *R1	1.5	V
**Hyperthermophilic bacteria**	*Aquifex aeolicus *VF5	2.1	
	*Thermotoga maritima *MSB8	3.0	V
**Archae Euryarchaeota**	*Methanococcus jannaschii *DSM2661	1.8	M
	*Pyrococcus furiosus *DSM3638	2.0	M
	*Archaeoglobus fulgidus *DSM4304	2.1	
	*Thermoplasma acidophilum *DSM1728	1.5	
	*Methanosarcina acetivorans *C2A	1.3	V
	*Methanosarcina mazei *Goe1	1.3	
	*Pyrococcus abyssi*	2.1	
**Archae Crenarchaeota**	*Aeropyrum pernix *K1	2.0	
	*Sulfolobus solfotaricus *P2	1.6	
	*Pyrobaculum aerophilum *IM2	1.7	

### Description of the developed equations

#### Minimal Insert Coverage (MIC)-equation

Since the generation of inserts for a genomic DNA library is a random process, a large part of the represented genes may be co-represented with other genes by one spot on the microarray. This complicates data interpretation since it introduces an uncertainty on which gene or genes are responsible for differential signals from these spots. The impact of differential expression of a specific gene on the observed difference of the signal from a spot will be larger when a larger part of the genome fragment in that spot is covered by that gene. Moreover, the larger the part of the insert that is covered by a specific gene, the larger the chance that differential signals for the spot can be attributed to that gene, and the higher the chance that differential expression levels from that gene result in a statistically significant differential signal on the array.

The MIC-equation anticipates to this effect by predicting the number of genes that are (at least partially) present on an insert *and *cover at least a predefined part of the insert (*DIC*). This predefined part is defined as a percentage of the total insert. E.g. if the insert size is 1000 base pairs and the predefined minimal insert coverage (*DIC*) is set at 50%, then at least 500 bp of that gene should be present on an insert to be considered as represented by the array. Genes smaller than the size of the predefined part of the insert, are considered as not represented on the array.

#### Gene Specific Information (GSI)-equation

**Figure 1:**
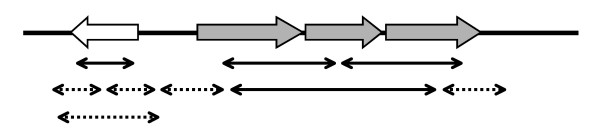
Schematic representation of the criteria that were applied to determine whether gene specific information is generated by a specific insert. The upper line represents a genome fragment in which the block arrows represent genes. Arrows with a gray filling belong to the same transcription unit. The thinner lines represent possible locations of the inserts. The dashed lines represent inserts for which no gene specific information can be generated, since they contain genomic material that possibly belongs to another transcription unit.

Information on differential expression of a gene can only be quantitative and specific for that gene if it originates from a spot that only represents genes from a single transcription unit, assuming that all genes within one transcription unit are equally expressed. This was the requirement that was set for a gene to be considered represented according to the gene specific information (GSI) equation. The criteria for spots that could generate gene specific information are visualized in Fig. 1. One of the variables in the GSI-equation, the minimal overlap (*O*_*mf*_), allows one to set the minimal number of base pairs that are required for identification of a specific gene or transcript on an insert on the clone-based array.

### Dataset preparation

Fifteen prokaryotes from various genera were selected as model species (Table 1). Genome data from these microorganisms were used for the generation of species-specific values for the expected fraction of represented genes as a function of the genome size (*GS*), number of clones (*N*), insert size (*IS*), and either *DIC *or *O*_*mf*_. Coordinates from all annotated genes from these organisms were obtained from GenBank, and were used to determine the gene sizes. In addition, information was obtained on the start and stop coordinates from the transcription unit to which the gene belongs, and the position of the gene in this transcription unit. It was assumed that transcription units start at the first base pair of the first gene and finish at the last base pair of the last gene. This information was generated by the combination of intergenic region based transcription unit predictions, generated by Moreno-Hagelsieb and Collado-Vides [6], with gene coordinates from GenBank.

The genome size (*GS*) could be included as a fictitious variable in the datasets, since not the species-specific genome size, but the species-specific gene size distribution and genome organization in genes and transcription units were relevant.

It was assumed that each possible genome fragment of the size of the insert size (*IS*) has an equal chance of being represented. To achieve this, fragments should be generated by physical fragmentation, and not by the use of endonucleases.

#### Dataset preparation for the fitting procedure for the MIC-equation

For each model organism, the fraction of the represented genes was determined for multiple combinations of the number of clones (*N*), fictitious genome size (*GS*), the insert size (*IS*), and the minimal insert coverage (*DIC*) in the ranges depicted in Table 2. In total, 140 different combinations of values for these variables were tested per strain. This was performed by first calculating the probability value of being represented per gene, and subsequently calculating the average of the probability values from all genes from the organism.

**Table 2:** Overview of the variables that were used for the model datasets on which the MIC- and the GSI-equation are based. Multiple combinations of the mentioned values were applied.

**Variable**	**Values**
*N*	500; 1500; 2500; 3500; 4500; 5500; 6500; 7500; 8500; 9500
*IS*	100; 300; 500; 700; 900; 1100; 1300; 1500; 2100; 2700; 3000
*GS*	0.5; 1.5; 2.5; 3.5; 4.5; 5.5; 6.5; 7.5; 8.5; 9.5
*O*_*mf*_	50; 100; 150; 200; 250; 300; 350; 400; 450
*DIC*	10; 20; 30; 40; 50; 60; 70; 80; 90

The following formulas were developed for the calculation of the probability value per gene:

Omv=IS⋅DIC100     (1)
 MathType@MTEF@5@5@+=feaafeart1ev1aaatCvAUfKttLearuWrP9MDH5MBPbIqV92AaeXatLxBI9gBaebbnrfifHhDYfgasaacH8akY=wiFfYdH8Gipec8Eeeu0xXdbba9frFj0=OqFfea0dXdd9vqai=hGuQ8kuc9pgc9s8qqaq=dirpe0xb9q8qiLsFr0=vr0=vr0dc8meaabaqaciaacaGaaeqabaqabeGadaaakeaacqWGpbWtdaWgaaWcbaGaemyBa0MaemODayhabeaakiabg2da9maalaaabaGaemysaKKaem4uamLaeyyXICTaemiraqKaemysaKKaem4qameabaGaeGymaeJaeGimaaJaeGimaadaaiaaxMaacaWLjaGaeiikaGIaeGymaeJaeiykaKcaaa@4077@

Gene>Omv⇒p=1−(1−Gene+1+IS−2⋅OmvGS)N     (2)
 MathType@MTEF@5@5@+=feaafeart1ev1aaatCvAUfKttLearuWrP9MDH5MBPbIqV92AaeXatLxBI9gBaebbnrfifHhDYfgasaacH8akY=wiFfYdH8Gipec8Eeeu0xXdbba9frFj0=OqFfea0dXdd9vqai=hGuQ8kuc9pgc9s8qqaq=dirpe0xb9q8qiLsFr0=vr0=vr0dc8meaabaqaciaacaGaaeqabaqabeGadaaakeaacqWGhbWrcqWGLbqzcqWGUbGBcqWGLbqzcqGH+aGpcqWGpbWtdaWgaaWcbaGaemyBa0MaemODayhabeaakiabgkDiElabdchaWjabg2da9iabigdaXiabgkHiTmaabmaabaGaeGymaeJaeyOeI0YaaSaaaeaacqWGhbWrcqWGLbqzcqWGUbGBcqWGLbqzcqGHRaWkcqaIXaqmcqGHRaWkcqWGjbqscqWGtbWucqGHsislcqaIYaGmcqGHflY1cqWGpbWtdaWgaaWcbaGaemyBa0MaemODayhabeaaaOqaaiabdEeahjabdofatbaaaiaawIcacaGLPaaadaahaaWcbeqaaiabd6eaobaakiaaxMaacaWLjaGaeiikaGIaeGOmaiJaeiykaKcaaa@5B31@

*Gene *<*O*_*mv *_⇒ *p *= 0     (3)

#### Dataset preparation for the fittimg procedure for the GSI-equation

For each organism, the fraction of genes for which specific information could be generated was determined for 114 different combinations of the number of clones (*N*), fictitious genome size (*GS*), the insert size (*IS*), and the minimal required overlap (*O*_*mf*_) in the ranges depicted in Table 2. The represented fraction was determined by taking the average of the probability values per gene. Formulas were developed that describe different situations with respect to the localization and organization of the gene of interest on the insert (eq 4 – 15)

Formulas that were developed to determine the probability value for genes that are transcribed into a single gene transcript:

Gene≥IS⇒p=1−(1−Gene+1−ISGS)N     (4)
 MathType@MTEF@5@5@+=feaafeart1ev1aaatCvAUfKttLearuWrP9MDH5MBPbIqV92AaeXatLxBI9gBaebbnrfifHhDYfgasaacH8akY=wiFfYdH8Gipec8Eeeu0xXdbba9frFj0=OqFfea0dXdd9vqai=hGuQ8kuc9pgc9s8qqaq=dirpe0xb9q8qiLsFr0=vr0=vr0dc8meaabaqaciaacaGaaeqabaqabeGadaaakeaacqWGhbWrcqWGLbqzcqWGUbGBcqWGLbqzcqGHLjYScqWGjbqscqWGtbWucqGHshI3cqWGWbaCcqGH9aqpcqaIXaqmcqGHsisldaqadaqaaiabigdaXiabgkHiTmaalaaabaGaem4raCKaemyzauMaemOBa4MaemyzauMaey4kaSIaeGymaeJaeyOeI0IaemysaKKaem4uamfabaGaem4raCKaem4uamfaaaGaayjkaiaawMcaamaaCaaaleqabaGaemOta4eaaOGaaCzcaiaaxMaacqGGOaakcqaI0aancqGGPaqkaaa@51B5@

*Gene *<*IS *⇒ *p *= 0     (5)

Formulas that were developed to determine the probability value for genes that are at the beginning of a transcription unit:

*BP*_*e *_≤ *IS *- *O*_*mf *_⇒ *O*_*e *_= *IS *- *BP*_*e *_    (6)

*BP*_*e *_> *IS *- *O*_*mf *_⇒ *O*_*e *_= *O*_*mf *_    (7)

BPe+Gene>IS⇒p=1−(1−Gene+1−OeGS)N     (8)
 MathType@MTEF@5@5@+=feaafeart1ev1aaatCvAUfKttLearuWrP9MDH5MBPbIqV92AaeXatLxBI9gBaebbnrfifHhDYfgasaacH8akY=wiFfYdH8Gipec8Eeeu0xXdbba9frFj0=OqFfea0dXdd9vqai=hGuQ8kuc9pgc9s8qqaq=dirpe0xb9q8qiLsFr0=vr0=vr0dc8meaabaqaciaacaGaaeqabaqabeGadaaakeaacqWGcbGqcqWGqbaudaWgaaWcbaGaemyzaugabeaakiabgUcaRiabdEeahjabdwgaLjabd6gaUjabdwgaLjabg6da+iabdMeajjabdofatjabgkDiElabdchaWjabg2da9iabigdaXiabgkHiTmaabmaabaGaeGymaeJaeyOeI0YaaSaaaeaacqWGhbWrcqWGLbqzcqWGUbGBcqWGLbqzcqGHRaWkcqaIXaqmcqGHsislcqWGpbWtdaWgaaWcbaGaemyzaugabeaaaOqaaiabdEeahjabdofatbaaaiaawIcacaGLPaaadaahaaWcbeqaaiabd6eaobaakiaaxMaacaWLjaGaeiikaGIaeGioaGJaeiykaKcaaa@5606@

*BP*_*e *_+ *Gene *<*IS *⇒ *p *= 0     (9)

Formulas that were developed to determine the probability value for genes that are flanked at both sides by other genes that belong to the same transcription unit:

*BP*_*b *_≤ *IS *- *O*_*mf *_⇒ *O*_*b *_= *IS *- *BP*_*b *_    (10)

*BP*_*b *_> *IS *- *O*_*mf *_⇒ *O*_*b *_= *O*_*mf *_    (11)

*BP*_*e *_≤ *IS *- *O*_*mf *_⇒ *O*_*e *_= *IS *- *BP*_*e *_    (12)

*BP*_*e *_> *IS *- *O*_*mf *_⇒ *O*_*e *_= *O*_*mf *_    (13)

BPb+BPe+Gene>IS⇒p=1−(1−Gene+1+IS−Ob−OeGS)N     (14)
 MathType@MTEF@5@5@+=feaafeart1ev1aaatCvAUfKttLearuWrP9MDH5MBPbIqV92AaeXatLxBI9gBaebbnrfifHhDYfgasaacH8akY=wiFfYdH8Gipec8Eeeu0xXdbba9frFj0=OqFfea0dXdd9vqai=hGuQ8kuc9pgc9s8qqaq=dirpe0xb9q8qiLsFr0=vr0=vr0dc8meaabaqaciaacaGaaeqabaqabeGadaaakeaacqWGcbGqcqWGqbaudaWgaaWcbaGaemOyaigabeaakiabgUcaRiabdkeacjabdcfaqnaaBaaaleaacqWGLbqzaeqaaOGaey4kaSIaem4raCKaemyzauMaemOBa4MaemyzauMaeyOpa4JaemysaKKaem4uamLaeyO0H4TaemiCaaNaeyypa0JaeGymaeJaeyOeI0YaaeWaaeaacqaIXaqmcqGHsisldaWcaaqaaiabdEeahjabdwgaLjabd6gaUjabdwgaLjabgUcaRiabigdaXiabgUcaRiabdMeajjabdofatjabgkHiTiabd+eapnaaBaaaleaacqWGIbGyaeqaaOGaeyOeI0Iaem4ta80aaSbaaSqaaiabdwgaLbqabaaakeaacqWGhbWrcqWGtbWuaaaacaGLOaGaayzkaaWaaWbaaSqabeaacqWGobGtaaGccaWLjaGaaCzcaiabcIcaOiabigdaXiabisda0iabcMcaPaaa@624C@

*BP*_*b *_+ *BP*_*e *_+ *Gene *> *IS *⇒ *p *= 0     (15)

### Models and fits

The datasets with the expected fractions of represented genes for the various combinations of parameters as presented in the previous section functioned as template for the fitting of the predictive equation for *MIC *and *GSI*.

#### MIC equation

From equation 2, which was used to determine the probability value per gene, it became apparent that organism-dependent gene size distribution influenced the expected number of represented genes on a clone based array. These organism dependent differences were neglected for the preparation of the MIC equation, which proofed to be justified when validating the MIC-equation (see validation section).

A polynome was developed as MIC model. In the polynome all variables were present in first and second order and in cross terms between two variables. Because of a high expected correlation between *IS *and *DIC *(based on equation 2), this relation was extended with a second order term composed of *IS *and *DIC*, resulting in:

*p*_*MIC *_= *a *+ *b*_1_·*DIC *+ *b*_2_·*DIC*^2 ^+ *c*_1_·*N *+ *c*_2_·*N*^2 ^+ *d*_1_·*GS *+ *d*_2_·*GS*^2 ^+ *e*_1_·*IS *+ *e*_2_·*IS*^2 ^+ *f*·*DIC*·*IS *+ *g*·*DIC*·*N *+ *h*·*DIC*·*GS *+ *i*·*IS*·*N *+ *j*·*IS*·*GS *+ *k*·*GS*·*N *+ *l*·(*IS*·*DIC*)^2 ^    (16)

The model datasets for the 15 model species were used together in the regression procedure to estimate the parameters in the MIC model. Linear regression using a standard least squares algorithm (fminsearch) provided by Matlab (The MathWorks) was applied to search the parameters that minimize the sum of squares (SSQ) defined as:

*SSQ *= ∑(*p*_*MIC*,exp _- *p*_*MIC*,mod_)^2 ^    (17)

The resulting parameters are presented in Table 3. The average absolute deviation of the MIC equation from the model dataset was 0.0517.

**Table 3:** Values for the parameters in the MIC- and the GSI-equation.

**parameter**	**MIC equation**	**GSI equation**	**GSI equation**
		***q***	***r***
***a***	4.85E-01	0.544	0
***b_1_***	2.54E-03	*	*
***b_2_***	-1.51E-05	-4.26E-08	-3.05E-07
***c_1_***	1.27E-04	6.13E-05	1.46E-05
***c_2_***	-5.22E-09	0	-1.96E-09
***d_1_***	-1.22E-01	-7.84E-02	-1.06E-02
***d_2_***	3.42E-03	3.31E-03	3.23E-04
***e_1_***	3.95E-04	-5.36E-04	2.08E-04
***e_2_***	-9.57E-08	9.73E-08	-4.62E-08
***f***	-9.85E-06	1.69E-08	3.42E-08
***g***	-4.61E-07	*	*
***h***	3.25E-04	2.55E-06	4.12E-06
***I***	-1.69E-08	-2.22E-08	5.47E-09
***j***	2.01E-05	2.42E-05	-6.04E-06
***k***	2.26E-06	-1.76E-06	1.30E-06
***l***	2.60E-11	*	*

ad *: this parameter is not present in the GSI equation

#### GSI equation

From the model datasets for the GSI equation it appeared that an organism dependent variable had a strong influence on the calculated number of represented genes (results not shown). Analysis revealed a positive correlation between the number of represented genes and the species-dependent average number of genes per transcription unit, *R*. *R *was determined by dividing the total number of genes (GenBank) by the total number of predicted transcription units [6] (Table 1).

Starting-point for the GSI model was a second order polynome for all variables, extended with the cross terms between two variables. A set of parameters was estimated for each individual model species (results not shown). Parts which appeared to contribute less than 1% to *p*_*GSI *_were not included, which resulted in the following relation:

*p*_*GSI *_= *a *+ *b*_2_·*O*_*mf*_^2 ^+ *c*_1_·*N *+ *c*_2_·*N*^2 ^+ *d*_1_·*GS *+ *d*_2_·*GS*^2 ^+ *e*_1_·*IS *+ *e*_2_·*IS*^2 ^+ *f*·*O*_*mf*_·*IS *+ *h*·*O*_*mf*_·*GS *+ *i*·*IS*·*N *+ *j*·*IS*·*GS *+ *k*·*GS*·*N *    (18)

For each prokaryote a set of parameters was obtained by minimizing the SSQ, equivalent to equation 17. The average absolute deviation of the GSI equation from the model datasets was 0.0258.

In order to obtain one generic equation for the organism specific relations for *p*_*GSI*_, the species specific values for the parameters (*a *- *k*) in equation 18 were related to the species related variable *R *by a linear relation:

*parameter*(*a *- *k*) = *q *+ *r*·*R *    (19)

in which *R *is species specific (Table 1). Since no dependency of *a *with *R *could be established, *a *was set at the average of all individual *a *values: 0.544. With this value the polynome was fitted again, and the final relations between the other parameters and *R *were determined (Table 3).

### Validations

**Figure 2:**
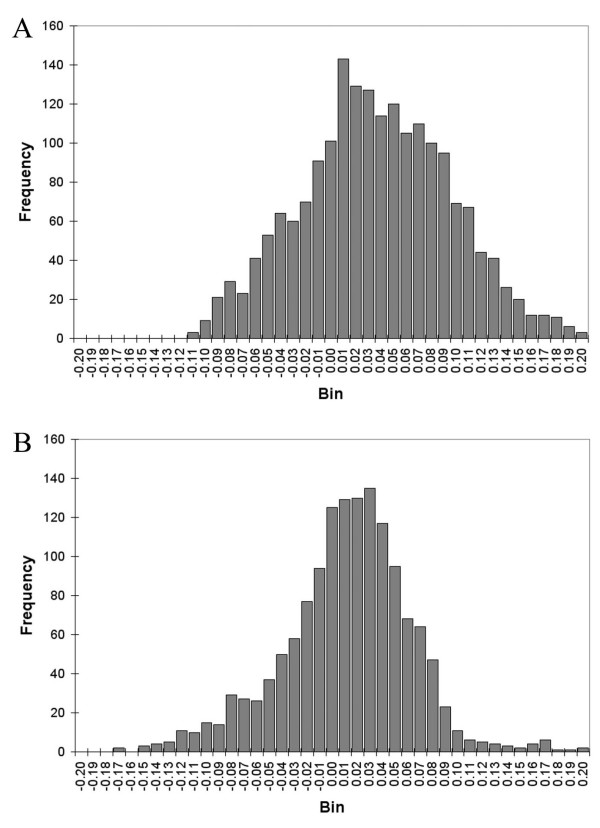
Histogram representations of the residuals from the validation of the MIC-equation (A) and the GSI-equation (B).

In order to validate the MIC- and the GSI-equation, datasets were generated (as previously described in the "dataset preparation" section) for ten validation species (Table 1). Represented gene fractions were calculated per species for all possible combinations for the variables as presented in Table 4 and distracted from the values as they were predicted by MIC- and the GSI-equations 16 and 18, respectively. The distributions of the residuals, i.e. the difference between predicted and the calculated fraction, for both equations are presented as histograms in Figures 2a and 2b. The residual distributions of both the MIC- and the GSI-equation approach the normal distribution with a slight tendency to underestimate the fraction of represented genes (Fig. 2a and 2b). Moreover, in Table 5 the reliability of the formulas is depicted as the fraction of predictions that differ less than 0.01, 0.05 and 0.10 from the real values. It should be noted that the indicated reliabilities relate to the range of variables as depicted in Table 4.

**Table 4:** Overview of the variables and the values used for these variables that were used for the datasets that were used for the validation of the MIC- and the GSI-equation. All possible combinations of the mentioned values were tested.

**Variable**	**Values**
N	1000; 4000; 7000; 10000
*IS*	100; 500; 1000; 1500; 2000
*GS*	1; 3; 5; 7
*DIC*	25; 50; 75
*O*_*mf*_	100; 200

**Table 5:** Reliability of the MIC- and the GSI-equation, depicted as the fraction of predictions that differ less than 0.01, 0.05 or 0.10 from the real values, for the validation sets defined in Table 4.

Abs (Δ predicted vs. real)	Fraction for MIC-equation	Fraction for GSI-equation
< 0.01	0.19	0.24
< 0.05	0.58	0.73
< 0.10	0.87	0.95

Deviations between the predicted fractions by the MIC-equation and the true values as they were determined for the validation species are mainly to be attributed to species-specific gene size distribution. In order to obtain one generic equation, and based on the accuracy of the equation in its current form (Table 5), it was decided not to include a species-specific variable.

### Prediction of the optimum value for the insert size (*IS*)

Whereas an increase in *N *will always have a positive contribution to the fraction of represented genes, and an increase in *GS*, *O*_*mf*_, and *MIC *a negative contribution, there may be an optimum *IS *that depends on the values of the other variables. This optimum can be estimated by differentiation of equation 16 and 18 to *IS *(d*p*/d*IS*).

For the determination of the optimal value for *IS *for the MIC-approach this results in the following equation:

dpMICdIS=e1+2e2⋅IS+f⋅DIC+i⋅N+j⋅GS+2⋅l⋅DIC2⋅IS=0⇒ISMIC−opt=−e1−f⋅DIC−i⋅N−j⋅GS2e2+2l(DIC)2     (20)
 MathType@MTEF@5@5@+=feaafeart1ev1aaatCvAUfKttLearuWrP9MDH5MBPbIqV92AaeXatLxBI9gBaebbnrfifHhDYfgasaacH8akY=wiFfYdH8Gipec8Eeeu0xXdbba9frFj0=OqFfea0dXdd9vqai=hGuQ8kuc9pgc9s8qqaq=dirpe0xb9q8qiLsFr0=vr0=vr0dc8meaabaqaciaacaGaaeqabaqabeGadaaakeaafaqaaeGabaaabaWaaSaaaeaacqWGKbazcqWGWbaCdaWgaaWcbaGaemyta0KaemysaKKaem4qameabeaaaOqaaiabdsgaKjabdMeajjabdofatbaacqGH9aqpcqWGLbqzdaWgaaWcbaGaeGymaedabeaakiabgUcaRiabikdaYiabdwgaLnaaBaaaleaacqaIYaGmaeqaaOGaeyyXICTaemysaKKaem4uamLaey4kaSIaemOzayMaeyyXICTaemiraqKaemysaKKaem4qamKaey4kaSIaemyAaKMaeyyXICTaemOta4Kaey4kaSIaemOAaOMaeyyXICTaem4raCKaem4uamLaey4kaSIaeGOmaiJaeyyXICTaemiBaWMaeyyXICTaemiraqKaemysaKKaem4qam0aaWbaaSqabeaacqaIYaGmaaGccqGHflY1cqWGjbqscqWGtbWucqGH9aqpcqaIWaamcqGHshI3aeaacqWGjbqscqWGtbWudaWgaaWcbaGaemyta0KaemysaKKaem4qamKaeyOeI0Iaem4Ba8MaemiCaaNaemiDaqhabeaakiabg2da9maalaaabaGaeyOeI0Iaemyzau2aaSbaaSqaaiabigdaXaqabaGccqGHsislcqWGMbGzcqGHflY1cqWGebarcqWGjbqscqWGdbWqcqGHsislcqWGPbqAcqGHflY1cqWGobGtcqGHsislcqWGQbGAcqGHflY1cqWGhbWrcqWGtbWuaeaacqaIYaGmcqWGLbqzdaWgaaWcbaGaeGOmaidabeaakiabgUcaRiabikdaYiabdYgaSjabcIcaOiabdseaejabdMeajjabdoeadjabcMcaPmaaCaaaleqabaGaeGOmaidaaaaaaaGccaWLjaGaaCzcaiabcIcaOiabikdaYiabicdaWiabcMcaPaaa@A1A7@

For the determination of the optimal value for *IS *for the GSI-approach the equation is as follows:

dpGSIdIS=e1+2e2⋅IS+f⋅O+i⋅N+j⋅GS=0⇒ISGSI−opt=−e1−f⋅O−i⋅N−j⋅GS2e2     (21)
 MathType@MTEF@5@5@+=feaafeart1ev1aaatCvAUfKttLearuWrP9MDH5MBPbIqV92AaeXatLxBI9gBaebbnrfifHhDYfgasaacH8akY=wiFfYdH8Gipec8Eeeu0xXdbba9frFj0=OqFfea0dXdd9vqai=hGuQ8kuc9pgc9s8qqaq=dirpe0xb9q8qiLsFr0=vr0=vr0dc8meaabaqaciaacaGaaeqabaqabeGadaaakeaafaqaaeGabaaabaWaaSaaaeaacqWGKbazcqWGWbaCdaWgaaWcbaGaem4raCKaem4uamLaemysaKeabeaaaOqaaiabdsgaKjabdMeajjabdofatbaacqGH9aqpcqWGLbqzdaWgaaWcbaGaeGymaedabeaakiabgUcaRiabikdaYiabdwgaLnaaBaaaleaacqaIYaGmaeqaaOGaeyyXICTaemysaKKaem4uamLaey4kaSIaemOzayMaeyyXICTaem4ta8Kaey4kaSIaemyAaKMaeyyXICTaemOta4Kaey4kaSIaemOAaOMaeyyXICTaem4raCKaem4uamLaeyypa0JaeGimaaJaeyO0H4nabaGaemysaKKaem4uam1aaSbaaSqaaiabdEeahjabdofatjabdMeajjabgkHiTiabd+gaVjabdchaWjabdsha0bqabaGccqGH9aqpdaWcaaqaaiabgkHiTiabdwgaLnaaBaaaleaacqaIXaqmaeqaaOGaeyOeI0IaemOzayMaeyyXICTaem4ta8KaeyOeI0IaemyAaKMaeyyXICTaemOta4KaeyOeI0IaemOAaOMaeyyXICTaem4raCKaem4uamfabaGaeGOmaiJaemyzau2aaSbaaSqaaiabikdaYaqabaaaaaaakiaaxMaacaWLjaGaeiikaGIaeGOmaiJaeGymaeJaeiykaKcaaa@839D@

If the indicated values for *IS*_opt _are outside the range of 0 to 2000 bp (the range that was applied for validation of the models) no optimum can be identified within the boundaries of the model. In these cases small values of *IS *will give the best results.

### Influence of the average number of genes per transcription unit (*R*) on the predicted values

From the input variables for the MIC and GSI formulas, *N*, *IS*, *DIC *and *O*_*mf *_are user-defined, while *GS *and *R *have to be estimated for the specific organism. Whereas current techniques allow for rapid and accurate estimations of *GS *[7-9], the organism specific value for *R *is difficult to determine for species from which little sequence information is available.

*R *was determined for 73 prokaryotes from multiple genera, as previously described in the "models and fits" section (Table 1). For 61 of the 73 strains in this list, *R *was within the narrow range from 1.5 – 2.0. Moreover these data indicate that accurate estimations of *R *can be made, based on the genus of the organism, with an exception for the mollicutes, the hyperthermophilic bacteria and the euryarchaeota.

The effect of false estimations of *R *was studied by the generation of validation sets as defined in Table 4 with the exception that higher or lower values for *R *were applied. The resulting values from the GSI-equation were compared with the true values (Table 6). It appeared that an over- or underestimation of 0.2 on *R *had limited effects on the fraction of predictions that differ less than 0.1 from the real values from the validation dataset (0.90 vs. 0.95 for the exact value of *R*). While an overestimation of 0.3 still results in 88% of the predictions that differ less than 0.1 from the real value from the validation dataset. This percentage was 80% in case of an underestimation of the same size.

**Table 6:** Effect of false estimations of *R *on the fraction of predictions that differ less than 0.01, 0.05 or 0.10 from the real values, for the validation set defined in Table 4.

Applied value for *R*	Abs (Δ predicted vs. real)	Fraction
*R*	< 0.01	0.24
	< 0.05	0.73
	< 0.10	0.95
*R *- 0.1	< 0.01	0.17
	< 0.05	0.65
	< 0.10	0.95
*R *+ 0.1	< 0.01	0.24
	< 0.05	0.75
	< 0.10	0.94
*R *- 0.2	< 0.01	0.12
	< 0.05	0.55
	< 0.10	0.90
*R *+ 0.2	< 0.01	0.23
	< 0.05	0.69
	< 0.10	0.91
*R *- 0.3	< 0.01	0.10
	< 0.05	0.38
	< 0.10	0.81
*R *+ 0.3	< 0.01	0.21
	< 0.05	0.59
	< 0.10	0.88

## Application

**Figure 3:**
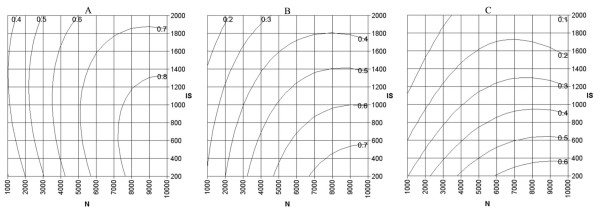
Contour plots of the predicted fractions of represented genes with a minimal insert coverage of 25% (A), 50% (B), or 75% (C) as a function of the number of clones (*N*) and the insert size (*IS*) for a prokaryote with a genome size of 4 Mbp. The predicted fractions are depicted in the plot on top of the lines by which they are represented.

**Figure 4:**
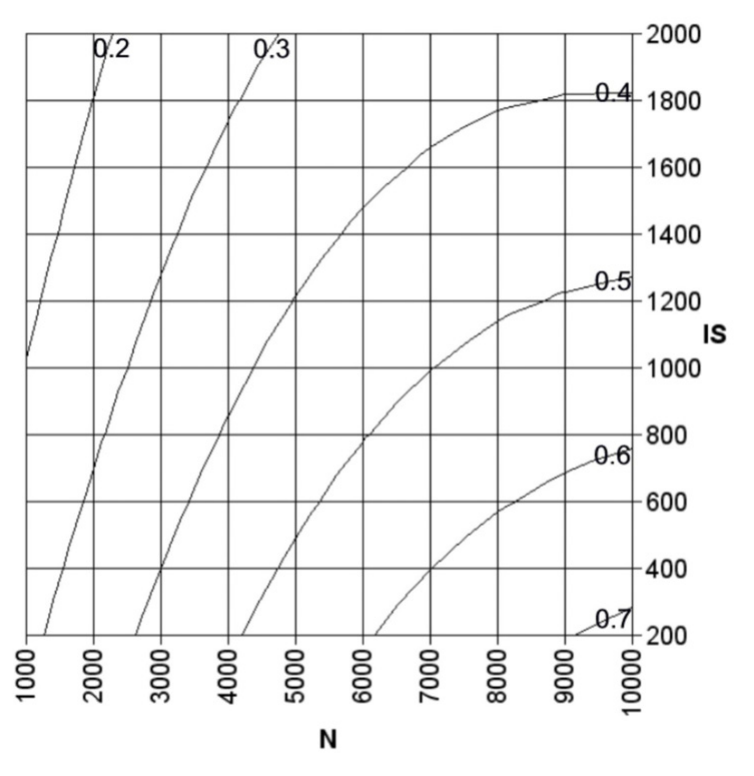
Contour plot of the predicted fraction of represented genes for which gene specific information could be generated as a function of the number of clones (*N*) and the insert size (*IS*) for a prokaryote with a genome size of 4 Mbp, an average number of genes per transcription unit (*R*) of 1.8, and a minimal overlap between the insert and the gene of 100 bp. The predicted fractions are depicted in the plot on top of the lines by which they are represented.

As an example for the applicability of the developed equations, the effect of different combinations of the number of clones and insert size was determined for a prokaryote with a genome size of 4 Mbp and an estimated value for *R *of 1.8 using equations 16 and 18. The effect of multiple combination of *N *and *IS *on p_MIC _was determined for minimal insert coverage (DIC) values of 25%, 50% and 75%. The results are depicted in the contour plots in figure 3a–3c. The predicted fractions of represented genes for which gene specific information could be generated (p_GSI_) with a minimal overlap between the insert and the gene of 100 bp is depicted in figure 4.

Plots like those depicted in figures 3a–c and 4 can be used to determine the preferred combination of the number of spots on the array and the insert size. If for instance the number of spots would be limited to 6000, an insert size of approximately 800 bp would be optimal with respect to the fraction of genes that are represented with a minimal insert coverage of 25% (Fig. 3a). From equation 20 this optimum appears to be 803 bp. With this combination of array parameters the predicted fraction of genes that cover at least 25% of the insert (which equals 803 × 0.25 = 201 bp) is 0.75 (eq. 16). Meanwhile the predicted fraction of genes for which gene specific information can be generated is 0.49 (eq. 18). If the specificity of the data is considered to be more important than the amount of represented genes, it is preferable to have an optimum value for p_MIC _for higher values of *DIC *(e.g. Fig. 3c) and a high value for p_GSI _(Fig. 4). These requirements are best fulfilled by combinations with low values for the insert size.

A Microsoft Excel fill in-spreadsheet that allows for calculations of pGSI, pMIC, and the optimal values for the insert size, is available as additional file with this paper [see Additional file 1].

## Discussion

Classical approaches for the construction of DNA libraries form a suitable base for the construction of clone-based microarrays. However, as the construction of these libraries is a random process, it is beforehand uncertain whether a gene or transcription unit will be uniquely represented on a separate insert on the array. Genome coverage by a DNA library is usually determined by calculating the expectation that each single nucleotide from that gene is present [3,4]. These formulas will overestimate the number of clones required when the library is to be used for the construction of a microarray, since for this purpose partial representation of a gene is sufficient for hybridization.

To our knowledge, Akopyants *et al*. were the first to estimate genome coverage at the gene level [5]. They predicted the fraction of represented genes using equation 22:

pAkopyants=1−(1−(average transcript size+insert size−2×required overlapgenome size))number of clones     (22)
 MathType@MTEF@5@5@+=feaafeart1ev1aaatCvAUfKttLearuWrP9MDH5MBPbIqV92AaeXatLxBI9gBaebbnrfifHhDYfgasaacH8akY=wiFfYdH8Gipec8Eeeu0xXdbba9frFj0=OqFfea0dXdd9vqai=hGuQ8kuc9pgc9s8qqaq=dirpe0xb9q8qiLsFr0=vr0=vr0dc8meaabaqaciaacaGaaeqabaqabeGadaaakeaacqWGWbaCdaWgaaWcbaGaemyqaeKaem4AaSMaem4Ba8MaemiCaaNaemyEaKNaemyyaeMaemOBa4MaemiDaqNaem4Camhabeaakiabg2da9iabigdaXiabgkHiTmaabmaabaGaeGymaeJaeyOeI0YaaeWaaeaadaWcaaqaaiabbggaHjabbAha2jabbwgaLjabbkhaYjabbggaHjabbEgaNjabbwgaLjabbccaGiabbsha0jabbkhaYjabbggaHjabb6gaUjabbohaZjabbogaJjabbkhaYjabbMgaPjabbchaWjabbsha0jabbccaGiabbohaZjabbMgaPjabbQha6jabbwgaLjabgUcaRiabbMgaPjabb6gaUjabbohaZjabbwgaLjabbkhaYjabbsha0jabbccaGiabbohaZjabbMgaPjabbQha6jabbwgaLjabgkHiTiabikdaYiabgEna0kabbkhaYjabbwgaLjabbghaXjabbwha1jabbMgaPjabbkhaYjabbwgaLjabbsgaKjabbccaGiabb+gaVjabbAha2jabbwgaLjabbkhaYjabbYgaSjabbggaHjabbchaWbqaaiabbEgaNjabbwgaLjabb6gaUjabb+gaVjabb2gaTjabbwgaLjabbccaGiabbohaZjabbMgaPjabbQha6jabbwgaLbaaaiaawIcacaGLPaaaaiaawIcacaGLPaaadaahaaWcbeqaaiabb6gaUjabbwha1jabb2gaTjabbkgaIjabbwgaLjabbkhaYjabbccaGiabb+gaVjabbAgaMjabbccaGiabbogaJjabbYgaSjabb+gaVjabb6gaUjabbwgaLjabbohaZbaakiaaxMaacaWLjaGaeiikaGIaeGOmaiJaeGOmaiJaeiykaKcaaa@B1F9@

An important variable in this formula is the average transcript size. However, use of this variable is not legitimate for this type of probability calculations since the average probability per gene (the required information) is not *per se *equal to the probability per average gene. When we validated the Akopyants formula on the same dataset that was applied for the validation of the MIC-equation, it appeared that 49% of the predictions deviated more than 0.1 from the real value (calculated as the average chance per gene), with a strong tendency to overestimation. The Akopyants formula therefore appears unreliable for calculating optimal library sizes

None of the previous formulas give insight in the fraction of genes for which gene specific information can be generated, while this is one of the most important features when one is interested in studying differential gene expression. The MIC-and GSI-equations that were developed in this study allow for good estimations of both the genome coverage at the gene level, and the fraction of genes for which gene specific transcription information can be generated.

Whereas the MIC-equation is rather straight-forward with respect to the input variables and interpretation, application of the GSI-equation requires the estimation of the average number of genes per transcription unit for an organism. Although a false estimation of this variable could lead to a wrong prediction of the represented fraction, Tables 1 and 6 indicate that this risk is limited.

The GSI-equation is partially based on operon predictions. For the development of the model and validation datasets we used log-likelihood based transcription unit predictions for adjacent pair of genes to be in the same operon [10]. This log-likelihood based prediction method is only applicable to organisms for which at least large parts of the genome have been sequenced, and will therefore not be useful when sequence data from array spots for which differential expression was identified, have to be interpreted. Nevertheless, good predictions can be made on whether or not genes that are co-represented in a single spot on the array belong to the same transcription unit. Strong indications can already be obtained from the physical organization of the DNA fragment of interest, like gene orientation and intergenic distance [6,11]. Other indications are the co-occurrence of genes with a joint function, and the conserved organization of homologous genes in other prokaryotes [11,12].

## Conclusion

The MIC- and GSI-equations that were developed in this study were based on genomes from 15 prokaryotes from different genera, and validated on the genomes of 10 other prokaryotes. These validations show that these equations form reliable tools for optimal design of prokaryotic clone-based microarrays within the ranges that were tested (Table 4), and that they are applicable to a broad range of prokaryotes. Therefore, these equations form a good basis for the design of microarrays for prokaryotes from which the genome sequence is not available.

## List of abbreviations

*BP*_*b *_number of base pairs within the operon in front of the specific gene [bp]

*BP*_*e *_number of base pairs within the operon behind the specific gene [bp]

*DIC *predefined minimal insert coverage, i.e. the minimal required representation of the gene on the insert [%]

*Gene *gene size [bp]

*GS *genome size [Mbp]

*IS *insert size [bp]

*IS*_*opt*-*MIC *_Optimal value of *IS *for the MIC equation [-]

*IS*_*opt*-*GSI *_Optimal value of *IS *for the GSI equation [-]

*N *number of clone [-]

*O*_*b *_overlap of the fragment and the beginning of the gene [bp]

*O*_*e *_overlap of fragment and the end of the gene [bp]

*O*_*mf *_minimal required overlap of the fragment and the gene (fixed) [bp]

*O*_*mv *_minimal required overlap of the fragment and the gene (variable) [bp]

*p *gene specific probability value [-]

*p*_*GSI *_predicted fraction of specifically represented genes [-]

*p*_*MIC *_predicted fraction of represented genes represented with a minimal insert coverage [-]

*R *average number of genes per transcription unit [-]

*SSQ *Residual Sum of Squares [-]

*a-l *parameters in MIC or GSI model [-]

## Authors' contributions

Bart Pieterse is responsible for the original idea behind this work and performed the statistical and validation procedures. Elisabeth Quirijns developed the MIC- and GSI-equations and performed the fitting procedures. Frank Schuren provided input on the construction and application of clone based microarrays. Mariët van der Werf focused on the interpretability and applicability of the developed equations. All authors read and approved the final manuscript.

## Supplementary Material

Additional File 1 The MIC- and GSI-equations (eq. 16 and 18), and the derived equations for prediction of the optimal values for *IS *(eq. 20 and 21), are available as a Microsoft Excel fill in spreadsheet. This spreadsheet can also be applied for the generation of contour plots in which the represented gene fractions are depicted as a function of the number of clones and the insert size. Click here for file
